# Current state of headache training within Canadian Neurology Residency program: a national survey

**DOI:** 10.1186/s12909-023-04571-z

**Published:** 2023-08-17

**Authors:** François Perreault, Suzanne Christie, Daniel Lelli, Susan Humphrey- Murto

**Affiliations:** 1 University of Ottawa, Ottawa, Canada; 2 Ottawa Headache Centre, Ottawa, Canada; 3 University of Ottawa Adult Neurology Residency Program, Ottawa, Canada; 4 Neurology and Otolaryngology, University of Ottawa, Ottawa, Canada; 5 Department of Medicine, University of Ottawa, Ottawa, Canada

**Keywords:** Headache, Residency training, Postgrad medical education

## Abstract

**Background:**

Headache disorders are the most common neurological disorders worldwide. Despite their widespread prevalence and importance, the topic of headache is inconsistently taught at both the undergraduate and postgraduate levels. The goal of this study is to establish a better picture of the current state of Headache Medicine (HM) training in Neurology postgraduate programs in Canada and describe the impact of the current pandemic on training in this domain.

**Methods:**

Online surveys were sent to senior residents of adult Neurology programs in Canada. We also conducted telephone interviews with Neurology Program Directors. Descriptive statistics were analyzed, and thematic analysis was used to review free text.

**Results:**

A total of 36 residents, and 3 Program Directors participated in the study. Most of the teaching in HM is done by headache specialists and general neurology faculty. Formal teaching is mainly given during academic half day. Most of the programs expose their residents to Onabotulinum toxin A injections and peripheral nerve blocks, but they don’t offer much formal teaching regarding these procedures. Residents consider HM teaching important and they would like to have more. They don’t feel comfortable performing interventional headache treatments, despite feeling this should be part of the skillset of a general neurologist.

**Conclusion:**

Our study is the first to establish the current state of headache teaching in post-graduate neurology programs as perceived by trainees and program directors in Canada. The current educational offerings leave residents feeling poorly prepared to manage headaches, including procedural interventions. There is a need to diversify the source of teaching, so the educational burden doesn’t lie mostly upon Headache specialists who are already in short supply. Neurology Residency programs need to adapt their curriculum to face the current need in HM.

**Supplementary Information:**

The online version contains supplementary material available at 10.1186/s12909-023-04571-z.

## Introduction

Headache disorders are the most common neurological disorders worldwide. Almost 3 billion people are affected by headache with tension-type headache and migraine being the most common [1]. An estimated 8.3% of Canadians in 2010–2011 were reported to have been diagnosed with migraine by a health professional [2]. Not only is headache the most common neurological disorder, but it is also among the most disabling disorders. It causes more disability-adjusted life years than all other neurological disorders combined. Migraine alone is the third leading cause of years lived with disability in people under the age of 50 [3]. It also represents almost a quarter of referrals to a neurologist in an outpatient setting [4].

Even though headache disorders represent a clear public health issue, there seems to be a discrepancy between the population needs and what is taught in medical school or in postgraduate programs. A recent survey sent to the Clinical Clerkship directors and/or curriculum deans of medical schools across the United States and Canada found that there was a lack of consistency and considerable variability on how Headache Medicine (HM) is taught. One of the barriers cited was the lack of available educational resources [5]. Over 200 Neurology and Family Medicine Residency Programs from the United States were surveyed, and they both felt the undergraduate curriculum did not adequately prepare residents for HM [6].

At the postgraduate level, similar concerns have been noted. A survey sent to the Neurology Program Directors (PDs) and the Neurology Chief Residents across the United States in 2016 revealed that programs do not offer adequate training in HM [7]. The Canadian Headache Society (CHS) offers annually a two- or three-day headache review course mostly for senior residents. This initiative seems to be well received by the residents, but it has never been evaluated.

More recently, the underrepresentation of headache lectures during neurology grand rounds was also highlighted [8]. In 2020, Canadian residency programs have shifted to the Competence by Design (CBD) curriculum. Representatives from all the Neurology Residency programs across Canada determined a list of clinical experiences required for successful completion of training for neurology residency. While some exposure to headache/pain patients is required, specialized HM clinics are not included as part of the required or recommended training experiences [9].

HM has evolved considerably in the last decade, with an increasing number of neurologists using interventional procedures, such as Onabotulinum toxin A injections and nerve blocks, to treat headaches [10]. However, a survey of Neurology PDs in the United States revealed that although exposure to these procedures is considered important, formal training is lacking [11]. Within the Canadian CBD curriculum, interventional procedures for HM are not mentioned in the list of specialized neurological procedures a graduating neurologist should be able to perform [12]. Onabutulinum toxin A injection clinics are only cited as an optional training experience [9].

It has been hypothesized that inadequate training in HM might in part explain why only one third of the patients are satisfied with their headache treatments [6, 13].

Since headache disorders represent one of the core neurological disorders cited by the Royal College of Physicians and Surgeons of Canada [14] and are clearly a public health issue, we believe Neurology Residency programs should better prepare their residents to care for these patients. Following the six-step approach in curriculum development established by Kern and al. [15], it is evident that a needs assessment is required. As cited earlier, Neurology PDs in the United States have been surveyed on the subject. However, data from Canadian Neurology Residency Training Programs is outdated and residents have never been surveyed. In order to inform a postgraduate headache curriculum, we require current data from learners and education leaders. Information seeking must also take into account the recent and ongoing pandemic, which has led to unprecedented changes in curriculum delivery [16].

Our goal is to establish a better understanding of the current state of HM training in Neurology postgraduate programs in Canada. We want to evaluate Neurology residents’ perceived comfort managing patients with headache and their desire for more training, including procedures. We also want to seek suggestions for program delivery in the setting of our current pandemic.

## Methods

We surveyed both Neurology residents and Neurology PDs across Canada. For the residents, we sent an online survey (see Supplementary material 1). PDs were included in order to obtain a more comprehensive perspective on headache education in their programs. Short telephone interviews were held with PDs (see Supplementary material 2), as the research team felt they would be more likely to respond to a short telephone interview than complete an on-line survey.

For the residents, we developed a self-administered survey based on the systematic approach described in the Association of Medical Education in Europe (AMEE) guide on developing questionnaires [17].

A literature review was conducted to determine if other relevant questionnaires were published and could be used or adapted for our goals. We used the Ovid database to conduct our review. With this information, the research team developed the questions of the survey and the interview guide. The development of the questions also drew upon the clinical and educational experience of the investigators involved in this project.

The survey and interviews sought to identify perceived gaps in knowledge and skills related to the diagnosis, management, and procedures relevant to care of patients living with headaches. The questionnaire gathered the following information: basic demographics; the presence of HM experts within their respective divisions; HM Fellowship programs or selectives/electives in HM; the current teaching methods used, including instruction for procedures; the residents’ and PDs’ opinions and suggestions for HM curriculum development and delivery modalities.

The questionnaires consisted of both multiple-choice questions and Likert-type response scales with several sections for comments. We sent the questions to a panel of experts in HM to ensure relevance and clarity of items: Dr. Ana Bradi, Dr. Vanessa Doyle, and Dr. Lucian Sitwell are all neurologists with clinical expertise in HM and they are all involved in the Headache Fellowship at the University of Ottawa.

The University of Ottawa is a bilingual university. Hence, the questionnaires were translated to French by a certified translator. We conducted pilot testing of the questionnaire in English with two Emergency physicians and in French with one surgeon to confirm clarity of the questions. All three physicians have a background in Medical Education.

The surveys were sent by email to the senior residents of every adult Neurology Residency program in Canada from postgraduate year three to five using Survey Monkey. We decided to exclude residents in postgraduate year one and two since those residents are often part of the Internal Medicine program and they would lack sufficient exposure to the Neurology training program curriculum to answer our questions.

To avoid survey fatigue, we conducted short telephone interviews using an abbreviated survey with the PDs. Notes were taken during the interviews.

We sent the web-based questionnaire link and cover letter by email to Neurology PDs and Neurology Program Administrators. The survey was then distributed to the senior residents within each program. A reminder was sent at week one and three after the initial e-mail to participants. We aimed to have a response rate of 60%; the survey was sent a total of three times. Each participant was eligible for a draw for an Amazon gift card of $125. The project was approved by the *Ottawa Health Science Network Research Ethics Board*. To understand the distribution of the data, we gathered the affiliated university of every responder for both questionnaires. This enabled the team to determine the percentage of universities that responded and if there was representation from across the country (i.e., East/West/Central and Quebec).

### Analysis

Descriptive statistics were used for quantitative data, and 2 research team members independently read the written comments to outline any themes. Since the questions in our survey were straightforward, analysis was considered to be a “manifest analysis”; we describe what is visible and obvious in the text and did not seek to extend understanding to the interpretive level [18].

## Results

### Resident’s survey

A total of 37 residents completed the survey. Since there are approximately 120 senior Adult Neurology residents across Canada this represents approximately 31%. One participant did not fill out the entire questionnaire and was not included in the study. Thus, a total of 36 questionnaires were analyzed. The resident participants represented 10 distinct neurology programs (of a possible 15) across the country as can be seen in Table 1. There were approximately equal numbers of PGY (postgraduate year)-3, 4, and 5 participants.

**Table 1:** Resident demographic characteristics

	Residents (*n* = 36)	
	n	%
Year of residency		
PGY3	13	36.1
PGY4	13	36.1
PGY5	10	27.8
Region of Canada^a^		
West	9	25.0
Central	14	38.9
Quebec	10	27.8
East	3	8.3

East- Includes New Brunswick and Nova Scotia

Central- includes Ontario

Quebec- Includes Quebec (primarily francophone population)

^a^West- includes British Columbia, Alberta, Saskatchewan and Manitoba

Almost half of the residents (44.4%) had taken an elective in HM within their program and/or as an external elective and 30.5% said that they would have if it was available at their home program. Only 16.7% are not considering taking an elective in HM.

Table 2 displays responses from residents. Since some programs had responses from more than one resident the data is also displayed for each of the 10 Adult Neurology residency programs. Of note, residents from the same program sometimes had discordant answers regarding the number of hours of teaching: one would answer “no official hours” and another one “more than 10 h”. Most of the programs have a rotation of subjects over a two-year cycle for their Academic Half-day. It is possible that some residents had not yet received planned lectures on HM or that such activities were not attended due to other reasons (post-call, vacation, etc.). Taking that into account, we decided to consider the highest number of hours reported when answers were different between residents coming from the same program.

**Table 2:** Adult Neurology residency program characteristic’s from total of 36 residents in 10 programs

	Residents (*n* = 36)		Neurology program (*n* = 10)	
	n	%	n	%
Does your Neurology residency program have a mandatory Headache Medicine rotation?				
Yes	5	13.9	2	20
No	31	86.1	8	80
Approximately, how many hours per year are dedicated to Headache Medicine during your formal teaching sessions?				
Academic Half day				
No official	2	5.6	0	0
0–5 h	13	36.1	3	30
5–10 h	9	25.0	1	10
> 10 h	12	33.3	6	60
Grand Rounds				
No official	9	25.0	5	50
0–5 h	12	33.3	3	30
5–10 h	1	2.8	1	10
> 10 h	2	5.6	1	10
No answer	12	33.3		
Journal Club				
No official	12	33.3	5	50
0–5 h	9	25.0	4	40
5–10 h	0	0.0	0	0
> 10 h	0	0.0	0	0
No answer	15	41.7		
Who usually teaches you Headache Medicine? Check all that apply				
Headache specialist faculty	27	75.0	8	80
General neurology faculty	17	47.2	8	80
Headache fellow	4	11.1	2	20
Senior resident	14	38.9	7	70
Nobody taught me Headache Medicine	1	2.8	0	0
In which context(s) do you usually encounter headache patients? Select all that apply				
Longitudinal clinic	32	88.9	10	100
Headache elective/selective	21	58.3	7	70
Inpatient ward	23	63.9	9	90
Outpatient clinic	29	80.6	10	100
Emergency department	36	100.0	10	100
I have never seen or rarely see a consultation for headache	1	2.8	0	0

Just under half of the programs have a specialized headache clinic, 20% have a mandatory rotation in HM and 20% offer a Fellowship in this domain. Most of the teaching is given by headache specialist faculty and fewer by general neurology faculty or senior residents. Residents encounter patients presenting with headaches in various settings, the most frequent being the emergency department, continuity, and outpatient clinics.

In terms of formal teaching sessions, 60% of the programs offer more than ten hours per year of academic half-day on HM. There are less than five hours of official teaching during Journal Club and Grand Rounds for most of the programs. The other way of teaching cited in the comments is the Headache Review course offered to all the programs across Canada annually by the Canadian Headache Society.

For interventional procedures, more than three quarters of the residents reported they were exposed at least once to Onabotulinum toxin A treatment and/or peripheral nerve block. Residents mostly learn these two procedures by practicing on real patients. Approximately 30% of the neurology programs do not offer lectures on these techniques. 60% of them offer practice on models for Onabotulinum toxin A injections and 40% for peripheral nerve block. The majority of the residents were not exposed to trigger point injections. Results are displayed in Table 3.

**Table 3:** Resident’s perspective on teaching of interventional procedures for Headache Medicine

	Residents (*n* = 36)		Neurology program (*n* = 10)	
	n	%	n	%
Were you ever exposed in your program to any of these procedures for the treatment of headache? Select all that apply				
Onabotulinum toxin A	31	86.1	9	90
Peripheral nerve block	27	75.0	9	90
Trigger point injection	7	19.4	5	50
If you have been exposed in your program to any of these procedures, how did you learn about the methods (including indications, contraindications, evidence, and adverse effects) of these procedures?				
Onabotulinum toxin A				
Lecture	18	50.0	7	70
Videos	8	22.2	6	60
Hands-on patient	24	66.7	9	90
Hands-on model	13	36.1	6	60
Other	2	5.6	2	20
None	6	16.7	1	10
Peripheral nerve block				
Lecture	14	38.9	7	70
Videos	8	22.2	7	70
Hands-on patient	24	66.7	8	80
Hands-on model	5	13.9	4	40
Other	2	5.6	2	20
None	7	19.4	0	0
Were you ever able to perform these procedures with or without supervision at some point during your training in your program? (Check if yes)				
Onabotulinum toxin A	17	47.2	8	80
Peripheral nerve block	23	63.9	9	90
Trigger point injection	3	8.3	3	30
Who trained or supervised you in the performance of these procedures? Check all that apply, and please provide at least one answer for each row				
Onabotulinum toxin A				
Headache specialist faculty	20	55.6	7	70
General neurology faculty	8	22.2	5	50
Headache fellow	1	2.8	1	10
Senior resident, Emergency department or Pain clinic faculty	0	0.0	0	0
Other	1	2.8	1	10
Peripheral nerve block				0
Headache specialist faculty	15	41.7	6	60
General neurology faculty	13	36.1	6	60
Headache fellow	2	5.6	1	10
Senior resident	5	13.9	3	30
Emergency department faculty	2	5.6	2	20
Pain clinic faculty	0	0.0	0	0
Other	1	2.8	1	10

Onabotulinum toxin A injections and peripheral nerve blocks are mostly taught by Headache specialist or general neurology faculty. Less than 50% of the residents had an opportunity to perform Onabotulinum toxin A injections with or without supervision. Approximately 63% of them were able to perform at least one peripheral nerve block. Comments from the residents regarding the teaching and supervision for these procedures: “Scant opportunity, [the faculty is] not eager to teach this»; “[I was] able to do a partial procedure once. Unfortunately, was not able to get the hands-on experience that I was hoping for.”; “This was on my emergency rotation rather than on Neurology.”; “I have read about botox injections on my own time and have watched them done in clinic—limited exposure.”; “I wish this was taught to us residents, as I know it is done by several staff neurologists.”.

94.4% of the residents consider headache as a moderately to extremely important public health issue and 88.9% of them consider it is moderately to extremely important to have a HM rotation during their residency training. Most of the residents consider it’s at least moderately important to receive teaching about interventional headache procedures and they think it is moderately to extremely important for a general neurologist to be able to perform these.

Table 4 displays resident’s level of comfort regarding the diagnosis and management of headaches. Residents perceive themselves as generally comfortable with the diagnosis and management of migraine and with selecting non-procedural treatment for headache. More than 50% of the residents said they are uncomfortable with the performance of interventional headache procedures. 72.2% and 86.1% of the residents are very to extremely interested in receiving more teaching respectively on non-procedural and procedural treatment related to HM. 94.4% of them would be likely to participate if there was a Canada-wide HM training program. In the comments section, many of the residents stated that a hybrid format allowing in-person practice for procedural skills would be the preferable delivery modality.

**Table 4:** Number and percentage of resident’s level of comfort regarding headache’s diagnosis and management

	Comfort level			
	Extremely comfortable	Comfortable	neutral	Uncomfortable
Diagnosis and management of migraine	16	19	0	1
Percentage (%)	44.4	52.8	0.0	2.8
Explaining the pathophysiology of migraine	6	20	8	2
Percentage (%)	16.7	55.6	22.2	5.6
Diagnosis and management of headaches other than migraine	3	21	9	3
Percentage (%)	8.3	58.3	25.0	8.3
Recognition of red flags for a patient presenting with headaches	19	16	1	0
Percentage (%)	52.8	44.4	2.8	0.0
Selecting appropriate preventive treatment for migraine based on published guidelines and position statements from recognized headache societies	9	22	4	1
Percentage (%)	25.0	61.1	11.1	2.8
Selecting appropriate acute treatment for migraine based on published guidelines and position statements from recognized headache societies?	11	19	4	2
Percentage (%)	30.6	52.8	11.1	5.6
The indications for interventional headache treatments (Onabotulinum toxin A, peripheral nerve injections…)	3	19	7	7
Percentage (%)	8.3	52.8	19.4	19.4
The performance of interventional headache treatments (Onabotulinum toxin A, peripheral nerve injections…)	2	9	5	20
Percentage (%)	5.6	25.0	13.9	55.6

Residents reported that the COVID-19 pandemic had an impact on HM teaching, mostly regarding procedural interventions. Some of them stated that their exposure to procedures were reduced due to the reduction of in-person clinics. That being said, other residents also mentioned that HM had been affected the same as the other areas of neurology training.

### Program director’s survey

Three PDs responded to our request for an interview, providing a response rate of 3/15 (see Supplemental material). They were all from the western region of Canada. Two of their programs have a specialized Headache clinic within their department and none of them offers a mandatory HM rotation. HM is mainly taught during Academic Half Day and not specifically during Journal Club or Grand rounds. Regarding procedural interventions, the PDs interviewed stated that their residents were mostly exposed to Onabotulinum toxin A injections and peripheral nerve blocks. These procedures are taught mainly through lectures and hands-on patient experience. These PDs consider neutral to moderately important to have a HM rotation as part of their residency curriculum. Two of the PDs consider it is very important to offer training in interventional procedures and one respondent suggested it’s “not at all important”. Regarding the desire to offer more training in procedures related to HM, answers varied from «not so interested» to «very interested». In the comments, two PDs stated that they consider their residents are already receiving enough training in this area. All three PDs would be very likely to send their residents to participate to a Canada-wide HM training program if available.

## Discussion

This Canadian survey study set out to gain a better understanding of the current state of HM training in Neurology postgraduate programs in Canada. We sought to understand resident perceived comfort level managing headaches, perceptions of adequacy of training including the use of procedures.

From the information we’ve gathered, residents perceive themselves as generally comfortable with certain aspects of HM such as the diagnosis and management of migraines and selecting non-procedural treatment for headaches. However, they do not express the same level of confidence when it comes to headaches other than migraines or to the indication and execution of interventional headache treatments. The reasons for this are not clear as residents describe extensive exposure to many patients with headaches in diverse clinical settings and most receive five to ten hours or more of formal teaching annually on HM. Formal training in procedures, however, appears to be lacking as described in a separate section below.

This finding is congruent with patient perceptions from the literature of being dissatisfied with care. Patients with headaches are primarily managed in an out-patient setting, and our results indicate approximately 90% of trainees see headaches in ambulatory clinics and approximately 60% have participated in a headache elective or selective which should support learning. Despite this they do not feel confident in managing patients with non-migraine headaches. Why? The management of headaches is complex, requiring individualized approaches and therefore may require a greater volume of patient exposure. An alternate explanation may be a lack of faculty expertise, comfort, and interest in managing these patients [19]. This would fit with our findings that most formal headache teaching is done by Headache specialist faculty. According to the residents, just under half of the teaching is done by General neurology faculty. Knowing that there are a limited number of Headache specialist faculty [17, 18], it would appear necessary for general neurology faculty to take an active role in headache teaching.

### A lack of teaching in procedural skills

Although 90% of programs provide the opportunity for trainees to actually perform procedures and 70% provide formalized training, a minority of residents feel comfortable performing the two most common procedures; Onabotulinum toxin A and peripheral nerve blocks. 

According to residents’ answers and comments, despite “exposure at the bedside”, teaching is inconsistent and infrequent. These data confirm what PDs from the US felt in the 2016 study [10]: residents don’t feel comfortable with the performance of these procedures, they want more training in this field and they think it’s important for a neurologist to be able to perform these. There is a need for more training in this area.

Even with adequate training, mastery of procedures requires practice and the number of procedures required to achieve procedural competence has been hotly debated [20]. Our study did not inquire about the actual number of procedures performed, but this is worthy of future studies.

According to our results, the majority of clinicians performing interventional procedures are headache specialists. Considering the high prevalence of headache disorders and the relatively low number of these specialists [21, 22], it would be reasonable to expect that general neurologists should be able to master these techniques. Thus, there is an unmet clinical need for physicians trained in interventional HM procedures. Since there are limited specialized faculty, alternative opportunities for training in injections techniques are required. Offering more formal teaching and hands-on practice regarding these procedures during residency using mannequins or simulation could represent a way to fill this gap. As an example, a study has already shown that training with mannequins for lumbar puncture can improve overall skill and may be superior to real-world unsupervised experience [23]. A similar approach could be used for the teaching of procedural intervention in HM. Alternatively, programs that provide training at a national level in order to train the trainers could be considered. This has been done successfully in Rheumatology for MSK US training. (CRUS course [24]).

### A general interest for more teaching in headache medicine

Most of the residents were very interested in receiving more teaching on procedural and non-procedural HM. Neurologists are considered to be the experts in HM. It is important for them to be comfortable with all HM aspects, including less common issues.

A Canada-wide HM program would be appreciated. The CHS, which is an external organization to residency programs and neurology departments, already offers to all Canadian Neurology residents a two- or three-day course every year on headaches [25]. This course is offered annually, and it has been mentioned by some of the interviewed residents as one of their formal teaching sessions on HM. Our questionnaire did not seek to evaluate the quality and appreciation of this initiative, and, to our knowledge, it has never been formally evaluated. According to the authors’ (FP) experience with this course, it is usually appreciated by the residents. Nevertheless, it mainly focuses on non-procedural HM and residents attend the course on a voluntary basis only. We do not know what percentage of residents take the course every year.

Since there is already an established national program for headaches, a next step would be a formal program evaluation to understand why it is not meeting the needs of the residents surveyed. We could also consider adding a component to teach procedures. The course is usually done in person, but it has changed to a virtual format in 2021 due to the COVID pandemic. Now that the pandemic situation has evolved, many of the residents stated in the comments section they would appreciate a hybrid format. This kind of approach could be beneficial since didactic material would be available online and in-person interaction would allow more time for small groups workshop and practice for interventional procedure. This flipped classroom approach has shown demonstrated benefits in the past [26] and it would be interesting to use it for HM. Interestingly, the American Headache Society has developed a program using this type of approach (REACH program [27]). No studies are available to our knowledge to explore the impact of this program, but it provides a model that could be adapted for the Canadian context. A similar approach with favorable outcomes has been used to teach neuroimmunology, which seems to be facing the similar challenges as HM [28].

There is a huge gap in learning procedures despite residents perceiving HM as highly relevant. New approaches need to be developed: a formal national headache teaching program with the use of simulation could meet this need.

### Results from program directors’ interview

Since we were only able to interview three Adult Neurology PDs, we can’t generalize any of the findings. This low response rate could partially be explained by the high amount of solicitation PDs can get to participate to study and the lack of interest in HM identified a few years ago within faculty members [19]. This could reflect a recognized stigma within the medical community towards HM [29].

### Strength, limitations and future research

To our knowledge, this study represents the first one to seek Canadian Adult Neurology Residents’ perspective on HM training. This gives a better picture of the current state of teaching on this subject at a postgraduate level in Canada. The survey was rigorously developed and pilot tested by experts in Medical Education and in HM. We think our sample is representative of the residents’ interest since less than 50% of them had done selective or elective rotations in HM. Although, we did not reach our ideal response rate of 60%, we do have representation from 10 of the 15 programs and representation from across the country and senior post-graduate years. Survey fatigue combined with a general low interest for HM might explained this lower response rate. As stated before, the response rate of three out of fifteen for the Adult Neurology PDs makes it difficult to generalize the findings.

Although this survey mainly represents the perspective of residents that were part of the prior non-CBD curriculum, the new Royal College CBD curriculum still does not require any mandatory exposure to headache medicine clinics nor any specific evaluation of HM competencies, hence is unlikely to change the status quo. This study has shown that there is a need to expand the teaching in HM, but this might not be feasible considering neurology residencies are of finite duration and the list of topics to cover ever expanding. Nevertheless, the underrepresentation of HM within neurology departments has been well documented [8, 19]. It would be interesting to evaluate the proportion of formal teaching hours for residents in HM, a very common issue, compared to other neurology topics.

Our study did not explore the reasons that residents still feel ill-prepared but we hope this work has reinvigorated the desire to improve HM training to address the burden of headache within society.

## Conclusion

Our study is the first to establish a better picture of the expressed and perceived needs of the Canadian Adult Neurology residents about HM training. Despite the actual clinical and teaching exposure to HM, residents still feel ill-prepared with many aspects of this field.

There is a need to diversify the source of the teaching so the burden does not rest solely upon Headache specialists who are already in short supply. There is a clear need from the residents to receive more formal teaching on procedural interventions, especially Onabotulinum toxin A injections and peripheral nerve blocks. There is also a general interest in receiving more teaching for all HM aspects. A hybrid Canadian-wide program using virtual and in-person teaching might offer a valued resource in the future.

### Supplementary Information

**Additional file 1:** **Supplemental Material 1.** Neurology resident survey on headache education.

**Additional file 2:** **Supplemental Material 2. **Neurology program director interview on headache education.

## Data Availability

All data generated or analysed during this study are included in this published article and its supplementary information files.
