# Supplementary material for: Current state of headache training within Canadian Neurology Residency program: a national survey
Source: BMC Med Educ. 2023 Aug 17;23:581. doi: 10.1186/s12909-023-04571-z (PMC10433594; doi:10.1186/s12909-023-04571-z)
Supplement: Supplementary file 1 — Additional file 1: Supplemental Material 1. Neurology resident survey on headache education. [file 12909_2023_4571_MOESM1_ESM.docx]

**Supplemental Material 1 - Neurology resident survey on headache education**

1. Select your year of residency

- PGY-3
- PGY-4
- PGY-5
- PGY-6 or more

1. Which University are you currently affiliated with?

- University of Alberta
- University of British Columbia
- University of Calgary
- Dalhousie University
- Université Laval
- McGill University
- Memorial University of Newfoundland
- University of Manitoba
- Université de Montréal
- University of Ottawa
- Queen’s University
- University of Saskatchewan
- Université de Sherbrooke
- University of Toronto
- Western University

1. Does the Neurology Department in which you are currently training have a Headache Medicine program? (i.e., specialized Headache Clinic)

- Yes
- No
- I don’t know
- Comments:

1. Does your university/department offer a Fellowship in Headache Medicine?

- Yes
- No
- I don’t know
- Comments

1. Does your Neurology residency program have a mandatory Headache Medicine rotation?

- Yes
- No
- I don’t know
- Comments:

1. Have you ever taken or are you scheduled to take an elective in Headache Medicine at some point during your residency? Select all that apply.

- Yes, at my home program
- Yes, as an external elective
- No, but I would have if it was available at my home program
- No, but I considered it
- No and I am not considering it
- Comments:

1. Approximately, how many hours per year are dedicated to Headache Medicine during your formal teaching sessions? (Please select one option for each column)

- For other, please list:

|  | Academic half day | Journal club | Grand rounds | Other |
| --- | --- | --- | --- | --- |
| No official hours are dedicated to Headache Medicine |  |  |  |  |
| 0-5 hours |  |  |  |  |
| 5-10 hours |  |  |  |  |
| >10 hours |  |  |  |  |

- Comments:

1. Who usually teaches you Headache Medicine? Check all that apply.

- Headache specialist faculty
- General neurology faculty
- Headache fellow
- Senior resident
- Nobody taught me Headache Medicine
- Other (please indicate)
- Comments:

1. In which context(s) do you usually encounter headache patients? Select all that apply.

- Continuity clinic/Longitudinal clinic
- Headache elective/selective
- Inpatient ward
- Outpatient clinic
- Emergency department
- I have never seen or rarely see a consultation for headache
- Other (please indicate)

1. Were you ever exposed in your program to any of these procedures for the treatment of headache? Select all that apply.

- Onabotulinum toxin A
- Peripheral nerve block
- Trigger point injection
- Comments:

1. If you have been exposed in your program to any of these procedures, how did you learn about the methods (including indications, contraindications, evidence, and adverse effects) of these procedures? Check all that apply and please provide at least one answer for each row.

|  | Lecture | Videos | Hands-on patient | Hands-on model | Other | None |
| --- | --- | --- | --- | --- | --- | --- |
| Onabotulinum toxin A |  |  |  |  |  |  |
| Peripheral nerve blocks |  |  |  |  |  |  |
| Trigger point injections |  |  |  |  |  |  |

- Please elaborate on any of the above, including other sources outside your program (e.g., Youtube video):
- For other, please list:
- Comments:

1. Were you ever able to perform these procedures with or without supervision at some point during your training in your program? (Yes/No question)

- Onabotulinum toxin A
- Peripheral nerve block
- Trigger point injection
- If yes to any of the above, please provide details:

1. Who trained or supervised you in the performance of these procedures? Check all that apply, and please provide at least one answer for each row.

|  | Headache specialist faculty | General neurology faculty | Headache fellow | Senior resident | Emergency department faculty | Pain Clinic faculty | Other | I have never performed this procedure |
| --- | --- | --- | --- | --- | --- | --- | --- | --- |
| Onabotulinum toxin A |  |  |  |  |  |  |  |  |
| Peripheral nerve blocks |  |  |  |  |  |  |  |  |
| Trigger point injections |  |  |  |  |  |  |  |  |

- For other, please list:
- Comments:

1. In your opinion, indicate the importance of these items. Please complete an item for each row

|  | Extremely important | Moderately important | Neutral | Not at all important |
| --- | --- | --- | --- | --- |
| How important is headache as a public health issue? |  |  |  |  |
| How important is it to have a Headache Medicine rotation during your residency? |  |  |  |  |
| How important is it to have instruction on interventional headache procedures (Onabotulinum toxin A, peripheral nerve injections...) |  |  |  |  |
| How important is it for a practicing general neurologist to be able to perform interventional headache procedures? (Onabotulinum toxin A, peripheral nerve injections...) |  |  |  |  |

- Comments:

1. Indicate how comfortable you are with these situations.

|  | **Extremely comfortable** | **Comfortable** | **Neutral** | **Slightly un-comfortable** | **Not at all comfortable** |
| --- | --- | --- | --- | --- | --- |
| The diagnosis and management of migraine? |  |  |  |  |  |
| Explaining the pathophysiology of migraine? |  |  |  |  |  |
| The diagnosis and management of headaches other than migraine? |  |  |  |  |  |
| The recognition of red flags for a patient presenting with headache? |  |  |  |  |  |
| Selecting appropriate preventive treatment for migraine based on published guidelines and position statements from recognized headache societies? |  |  |  |  |  |
| Selecting appropriate acute treatment for migraine based on published guidelines and position statements from recognized headache societies? |  |  |  |  |  |
| The indications for interventional headache treatments (Onabotulinum toxin A, peripheral nerve injections…)? |  |  |  |  |  |
| The performance of interventional headache treatments (Onabotulinum toxin A, peripheral nerve injections…)? |  |  |  |  |  |

- Comments:

1. Do you desire more training in non-procedural Headache Medicine?

- Extremely interrested……Somewhat interrested……Not at al interrested
- Please elaborate:

1. Do you desire more training in procedures related to Headache Medicine?

- Extremely interrested……Somewhat interrested……Not at al interrested
- Please elaborate:

1. If a Canada-wide Headache Medicine training program were offered, how likely would it be for you to participate?

- Very likely………………………not very likely
- Comments:

1. If a Canada-wide Headache Medicine training program were offered, what would be your preference for delivery? (e.g., in-person lecture, online lecture, etc.)

- Comments:

1. How has the COVID pandemic impacted training in Headache Medicine in your institution?

- Please elaborate:

1. Any comments or thoughts about headache education in general, and in light of the recent COVID pandemic? (Open question)
