# Supplementary material for: Current state of headache training within Canadian Neurology Residency program: a national survey
Source: BMC Med Educ. 2023 Aug 17;23:581. doi: 10.1186/s12909-023-04571-z (PMC10433594; doi:10.1186/s12909-023-04571-z)
Supplement: Supplementary file 2 — Additional file 2: Supplemental Material 2. Neurology program director interview on headache education. [file 12909_2023_4571_MOESM2_ESM.docx]

**Supplemental Material 2 - Neurology program director interview on headache education**

1. Which University are you currently affiliated with?

- University of Alberta
- University of British Columbia
- University of Calgary
- Dalhousie University
- Université Laval
- McGill University
- Memorial University of Newfoundland
- University of Manitoba
- Université de Montréal
- University of Ottawa
- Queen’s University
- University of Saskatchewan
- Université de Sherbrooke
- University of Toronto
- Western University

1. Does your Neurology Department have a Headache Medicine program? (i.e., specialized Headache Clinic)

- Yes
- No
- Comments:

1. Does your university/department offer a Fellowship in Headache Medicine?

- Yes
- No
  Comments

1. Does your neurology residency program have a mandatory Headache Medicine rotation?

- Yes
- No
- Comments:

1. Approximately, how many hours per year are dedicated to Headache Medicine during your formal teaching sessions? (Please select one option for each column)

- For other, please list:
- Comments:

|  | Academic half day | Journal club | Grand rounds | Other |
| --- | --- | --- | --- | --- |
| No official hours are dedicated to Headache Medicine |  |  |  |  |
| 0-5 hours |  |  |  |  |
| 5-10 hours |  |  |  |  |
| >10 hours |  |  |  |  |

- Please elaborate on other if applicable

1. Who usually teaches Headache Medicine to your residents?

- Headache specialist faculty
- General neurology faculty
- Headache fellow
- Senior resident
- Nobody is specifically teaching headache
- Other (please indicate)
- Comments:

1. In which context(s) do your residents usually encounter headache patients? Select all that apply.

- Continuity clinic/Longitudinal clinic
- Headache elective/selective
- Inpatient ward
- Outpatient clinic
- Emergency department
- Other (please indicate)

1. Are your residents ever exposed to any of these procedures for the treatment of headache?

- Onabotulinum toxin A
- Peripheral nerve block
- Trigger point injection
- I don’t know
- Comments:

1. If your residents have been exposed to any of these procedures, how did they learn or train to perform these procedures (including indications, contraindications, evidence, and adverse effects)? Check all that apply. And please provide at lead one answer for each row.

|  | Lecture | Videos | Hands-on patient | Hands-on model | Other | None |
| --- | --- | --- | --- | --- | --- | --- |
| Onabotulinum toxin A |  |  |  |  |  |  |
| Peripheral nerve blocks |  |  |  |  |  |  |
| Trigger point injections |  |  |  |  |  |  |

- Please elaborate on any of the above, including other sources outside your program (e.g., Youtube video):

1. Who trained or supervised your residents during the performance of these procedures? Check all that apply and please provide at least one answer for each row.

|  | Headache specialist faculty | General neurology faculty | Headache fellow | Senior resident | Emergency department faculty | Pain Clinic faculty | Other | I don’t know |
| --- | --- | --- | --- | --- | --- | --- | --- | --- |
| Onabotulinum toxin A |  |  |  |  |  |  |  |  |
| Peripheral nerve blocks |  |  |  |  |  |  |  |  |
| Trigger point injections |  |  |  |  |  |  |  |  |

- For other, please list:
- Comments:

1. When considering your program’s priorities, indicate how important are these items:

|  | Extremely important | Moderately important | Neutral | Not at all important |
| --- | --- | --- | --- | --- |
| How important is headache as a public health issue? |  |  |  |  |
| How important is it to have a Headache Medicine rotation as part of your residency curriculum? |  |  |  |  |
| How important is it for your program to train residents on interventional headache procedures (Onabotulinum toxin A, peripheral nerve injections...) |  |  |  |  |
| How important is it for a practicing general neurologist to be able to perform interventional headache procedures? (Onabotulinum toxin A, peripheral nerve injections...) |  |  |  |  |

- Comments:

1. Is there a desire for your program to offer more training in non-procedural Headache Medicine?

- Extremely interrested……Somewhat interrested……Not at al interrested
- Please elaborate:

1. Is there a desire for your program to offer more training in procedures related to Headache Medicine?

- Extremely interrested……Somewhat interrested……Not at al interrested
- Please elaborate:

1. Do you feel that your residents are adequately prepared to diagnose and treat headaches upon graduation?

- Please elaborate:

1. If a Canada-wide Headache Medicine training program were offered, how likely are you to send your residents to participate?

- Very likely………………………not very likely
- Comments:

1. If a Canada-wide Headache Medicine training program was offered, what would be your preferred method of delivery? (e.g., in-person lecture, online lecture, etc.)

- Comments:

1. How has the COVID pandemic impacted training in Headache Medicine in your institution?

- Please elaborate:

1. Any comments or thoughts about headache education in general, and in light of the recent COVID pandemic? (Open question)
